# A Coexisting Fungal-Bacterial Community Stabilizes Soil Decomposition Activity in a Microcosm Experiment

**DOI:** 10.1371/journal.pone.0080320

**Published:** 2013-11-18

**Authors:** Masayuki Ushio, Takeshi Miki, Teri C. Balser

**Affiliations:** 1 Center for Ecological Research, Kyoto University, Otsu, Shiga, Japan; 2 Department of Soil Science, University of Wisconsin-Madison, Madison, Wisconsin, United States of America; 3 Institute of Oceanography, National Taiwan University, Taipei, Taiwan; 4 Department of Soil and Water Science, University of Florida IFAS, Gainesville, Florida, United States of America; University of New South Wales, Australia

## Abstract

How diversity influences the stability of a community function is a major question in ecology. However, only limited empirical investigations of the diversity–stability relationship in soil microbial communities have been undertaken, despite the fundamental role of microbial communities in driving carbon and nutrient cycling in terrestrial ecosystems. In this study, we conducted a microcosm experiment to investigate the relationship between microbial diversity and stability of soil decomposition activities against changes in decomposition substrate quality by manipulating microbial community using selective biocides. We found that soil respiration rates and degradation enzyme activities by a coexisting fungal and bacterial community (a taxonomically diverse community) are more stable against changes in substrate quality (plant leaf materials) than those of a fungi-dominated or a bacteria-dominated community (less diverse community). Flexible changes in the microbial community composition and/or physiological state in the coexisting community against changes in substrate quality, as inferred by the soil lipid profile, may be the mechanism underlying this positive diversity–stability relationship. Our experiment demonstrated that the previously found positive diversity–stability relationship could also be valid in the soil microbial community. Our results also imply that the functional/taxonomic diversity and community ecology of soil microbes should be incorporated into the context of climate–ecosystem feedbacks. Changes in substrate quality, which could be induced by climate change, have impacts on decomposition process and carbon dioxide emission from soils, but such impacts may be attenuated by the functional diversity of soil microbial communities.

## Introduction

Soil microbial communities fundamentally drive decomposition processes by secreting extracellular enzymes, thereby playing potentially important roles in greenhouse gas emission, plant-soil interactions, nutrient cycling, and climate-ecosystem feedbacks [1], [2], [3], [4]. Because the performance of aboveground compartments (e.g., plants) in terrestrial ecosystems depend on nutrients that are mineralized by the decomposition process, changes in the decomposition activity of soil microbial communities have significant impacts on the dynamics of those aboveground compartments [5]. Therefore, little variation in the decomposition activity over an externally imposed change (or time period) would lead to the stable functioning of aboveground compartments of an ecosystem.

Stability can be defined as how little the rate of a concerned process varied over an externally imposed change or time period, although the term “stability” is a metaconcept that covers a wide range of different properties [6]. In recent decades, how diversity influences the stability of ecosystem functioning has become an increasingly important and urgent issue because of the increasing loss of biodiversity arising as a result of intensive human activities. With respect to the diversity–stability relationship, aboveground plant communities have been most intensively studied [7], [8], [9], while some studies have focused on aquatic ecosystems [10], [11]. Positive correlations in diversity–stability relationships (e.g. temporal variation in productivity is smaller in a species-rich community than in a species-poor community) have often been reported in previous studies [8], [12]. A large variation in the abundance of each species in species-rich communities is likely to stabilize community-level function by compensating for environmental fluctuations, and is thought to be the underlying mechanism of the positive correlations [8], [12], [13], [14].

However, it remains unclear whether positive relationships in aboveground plant communities are valid for the belowground part of ecosystems since evidence from belowground microbial communities remains limited [15] despite their importance for ecosystem function [5]. Previous studies have indicated that soil decomposition processes are generally controlled by the chemical quality of the decomposition substrates (mainly supplied as plant materials) such as the carbon to nitrogen ratio or lignin to nitrogen ratio [16]. In recent decades, however, enormous phylogenic diversity in soil microbial communities [17] and potential linkages between microbial diversity and decomposition processes [18] have been identified using molecular-based culture-independent techniques. Nonetheless, studies that focus on the significance of microbial diversity in the stability of decomposition activity are largely limited, except for theoretical studies [4].

The lack of studies on the diversity–stability relationship in soil microbial ecology is partly because of the difficulties associated with manipulating the composition of soil microbial communities. Some techniques such as the inoculum dilution technique have been applied to manipulate microbial community composition [19], but there is no perfect technique currently available. One of the most commonly applied techniques to manipulate microbial community composition is addition of a biocide to soil [20]. The technique cannot control the abundance of every individual species in the soil microbial community. However, it can roughly control the abundance and/or activity of the most fundamental functional groups of the soil microbial community (fungi and bacteria) by adding group-specific biocides such as fungicides and bactericides. The structure of the microbial community at the level of these fundamental groups (i.e., fungi and bacteria) is shown to change with environmental conditions in a regular way [21], [22] and is predicted to be responsible for functional stability [4]. Therefore, focusing on the taxonomically and physiologically distinct groups of soil microbial community will provide fundamental information about the significance of microbial taxonomic/functional diversity in the stability of soil decomposition activities.

In this study, we conducted a microcosm experiment to test the relationship between coarse-scale taxonomic diversity of microbes and the stability of decomposition activity. The term “stability” is used to indicate how little the decomposition activities varied over the changing quality of decomposition substrate in this microcosm experiment. We show that the decomposition activities of a coexisting fungal-bacterial community were more stably maintained against variations in plant—substrate quality than those of either a fungi-dominant or bacteria-dominant community, and that this could be accomplished by more flexible changes in the composition and/or physiological state of the coexisting community. Distilled water, a bactericide and fungicide were added to the A-horizon layer of a forest soil to create an intact (i.e., a coexisting fungal-bacterial community), a fungi-dominant, and a bacteria-dominant community, respectively. For decomposition substrates, powdered fresh leaves of two tree species mixed in various fixed proportions were added, and soil decomposition activity and soil lipid profile were measured after addition of the substrate.

## Materials and Methods

### Soil and leaf processing

Soil samples and plant leaves were collected in May 2010 from the Arboretum of the University of Wisconsin-Madison (43° 02′ N, 89° 25′ W). The soils were immediately brought back to the laboratory and sieved using a stainless sieve (2-mm mesh). Soil properties in the sampling site were as follows: pH in water  = 5.88±0.41, organic carbon content  = 3.30±0.85%, total nitrogen content  = 0.242±0.051%, carbon-to-nitrogen ration  = 13.6±1.1, and soil water content  = 30.5±5.9% (values after ± indicates standard deviation, N = 6). The soil samples were combined and mixed to produce homogeneous soil samples. For decomposition substrates, we collected fresh leaves of eastern hemlock (*Tsuga canadensis*) and sugar maple (*Acer saccharum*) from the same location as the soil sampling site. The chemical qualities of the leaves differ from each other (Table 1). The leaves were dried at 65°C, powdered, and mixed in fixed proportions (i.e., sugar maple:Eastern hemlock  = 0∶100, 25∶75, 50∶50, 75∶25, and 100∶0 [% dry weight basis]) to produce a variation in the quality of decomposition substrate. The composition of soil microbial community was manipulated by biocides; cycloheximide (Wako, CAS *66*-*81*-*9*) and oxytetracycline (Sigma, CAS *2058*-*46*-*0*) were used to stop fungal and bacterial activities, respectively [23]. Cycloheximide and oxytetracycline were dissolved in distilled water so that the final concentration of the biocides was 3 mg·g^-1^ dry soil. Hereafter, the fungicide (cycloheximide) and the bactericide (oxytetracycline) treatments are referred to as “bacterial community” and “fungal community”, respectively, while the distilled water treatment is refereed to as “coexisting community”. Influences of these treatments on soil microbial lipid profiles and their interpretation are shown in Supporting Information (see Result S1 and Fig. S1).

**Table 1:** Leaf chemistry of Eastern hemlock (*Tsuga canadensis*) and sugar maple (*Acer saccharum*) collected from the Arboretum of University of Wisconsin-Madison and used in the microcosm experiment in this study.

Chemical property	Eastern hemlock		Sugar maple	
C (%)	49.3	(0.09)	47.0	(0.08)
N (%)	1.36	(0.007)	2.32	(0.017)
C/N	36.2	(0.25)	20. 3	(0.12)
Total phenolics (mg·g^-1^)	65.4	(0.42)	124.4	(3.08)
Condensed tannins (mg·g^-1^)	1.82	(0.11)	0.87	(0.34)
Condensed tannins/N	1.33	(0.076)	0.38	(0.148)

Each value is the mean value (S.D.) of 3 repeated measurements.

### Microcosm experiment

One day after the addition of the biocides, the powdered and mixed leaves were added as external substrates, and the microbial decomposition activity was measured. Each incubation bin contained approximately 22 g of fresh soil (15 g of dry-soil equivalent), and 1 g of leaf powder was added as decomposition substrate. There were 4 replicates for each substrate-quality incubation for each of the three community types. Therefore, we had totally 60 samples for the microcosm experiment (i.e., 3 community types ×5 substrate quality ×4 replicates). During the incubation, the microcosms were maintained in the dark at a constant temperature of 20°C. The incubation bins were closed with lids, but there were 2 small holes to allow soil microbes to respire. Soil-water content was kept at 60% of water-holding capacity, which is almost identical to the field condition. One day (24 h) after the start of the incubation, 1 g and 3 g of fresh soil were collected for the analysis of enzyme activity and microbial lipid extraction, respectively. Since the activities were measured relatively soon after the biocide addition, one might speculate that the dead microbial biomass would have an effect on the assay results. However, it is less likely because amounts of the added substrate were much higher than total lipid abundance that is a proxy of microbial biomass (Fig. S1).

### Enzyme activity and soil respiration rate

The activities of 4 enzymes was measured spectrometrically by using commercially available methylumbelliferone (MUB)-linked substrates [24], [25], [26]. The activities of acid phosphatase, *N*-acetylglucosaminidase, β-d-glucosidase, and cellobiohydrolase were measured using 4-MUB-phosphate (CAS *3368*-*04*-*5*), 4-MUB-*N*-acetyl-β-d-glucosaminide (CAS *37067*-*30*-*4*), 4-MUB-β-glucopyranoside (CAS *18997*-*57*-*4*), and 4-MUB-d-cellobioside (CAS *72626*-*61*-*0*), respectively (all reagents were obtained from Sigma).

Soil suspensions were prepared by adding 1 g of fresh soil sample to 10 mL of 50 mM acetate buffer solution (pH 5.0). The tubes were shaken vigorously for 30 min. Four replicate measurements were made per sample for every assay. Using clipped tips, 200 µL of soil suspension was added to a 96-well black microplate, and then, 50 µL of substrate solution was added. Substrate and buffer auto fluorescence were checked by adding 50 µL of substrate solution and 50 µL of acetate buffer, respectively, to 200 µL of acetate buffer. Soil quenching was evaluated by adding 50 µL of MUB solution and 200 µL of soil suspension. Soil auto fluorescence was evaluated by adding 50 µL of acetate buffer and 200 µL of soil suspension. A standard curve was developed using MUB solution. The microplates were incubated at 20°C in the dark for 2 h. To stop the reaction, 10 µL of 1.0 M NaOH was added to each well. After 30 min, fluorescence was measured using 360-nm excitation and 460-nm emission filters with the Synergy 2 Multidetection Microplate Reader (Biotek Inc., Winooski, VT). Ideally, the time between adding NaOH and measuring fluorescence should be 1–2 min and constant among the samples [26], [27]. However, as the automatic plate reader was used in our experiment, it is better to wait more than 10 min to produce constant results [27]. After correcting for soil and substrate quenching and soil and substrate auto fluorescence, the activities were expressed as the concentration of degraded product per hour per soil (i.e., µmol·h^-1^·g^-1^).

Before measuring the soil respiration rate, the incubation bin was completely opened to exchange the inside gas with the ambient air. Then, a lid with 2 small holes was attached to the bin, and gas tubes were connected to the small holes. The concentration of carbon dioxide inside the bin was measured using a photoacoustic gas monitor (INNOVA 1412; LumaSense Technologies Inc., Santa Clara, CA). After the first measurement, the bin was closed and left undisturbed for 1 h, followed by the second measurement of the carbon dioxide concentration. The soil respiration rate was calculated as the difference between the first and the second measurements.

### Lipid analysis

Immediately after the soil sampling, a subset of the collected soil samples was homogenized, frozen, and freeze-dried for lipid analysis. Microbial lipid analysis was used to assess the microbial community composition of each freeze-dried soil sample. We extracted, purified, and identified lipids from microbial cell membranes by using a hybrid lipid extraction method as described previously [28]. Briefly, microbial lipids were extracted based on a modified Bligh and Dyer method [29]. After the extraction and purification, fatty acids were analyzed using a Hewlett-Packard 6890 Gas Chromatograph and peaks were identified using bacterial fatty acid standards and Sherlock peak identification software (MIDI Inc., Newark, DE). Then, we calculated the abundance of total lipid and each indicator lipid. Total lipid abundance was calculated as the sum of lipids whose chain length was from 10 to 20. The indicator lipids used for the calculations were as follows: Total bacterial biomass was estimated by the sum of the abundance of *i*14∶0, 15∶0, *i*15∶0, *a*15∶0, *i*16∶0, 17∶0, *i*17∶0, *a*17∶0, 16∶1ω7, *cy*17∶0, and *cy*19∶0 [30], [31]. Gram-positive bacteria were represented by the branched lipids, including *i*14∶0, *i*15∶0, *a*15∶0, *i*16∶0, *i*17∶0, and *a*17∶0 [32], whereas gram-negative bacteria were represented by the mono-saturated and cyclopropyl lipids, including 16∶1ω7, *cy*17∶0, and *cy*19∶0 [33], [34]. Fungal biomass was estimated by the abundance of 18∶2ω6,9 [30]. Actinomycetes were represented by 16∶0 10Me, 17∶0 10Me, and 19∶0 10Me. Arbuscular mycorrhizae, saprophytic fungi, and protozoa were represented by 16∶1ω5, 18∶2ω6,9, and 18∶3ω6,9,12, respectively. The fungi to bacteria ratio was calculated as 18∶2ω6,9/(sum of all bacterial lipids).

### Statistical analyses

For all analyses, the free statistical environment R was used [38]. Since we did not have an *a priori* hypothesis about the shape of the functions of the substrate-quality effects, additive models were chosen to investigate the relationships between the microbial decomposition activities and substrate quality. Decomposition activity was explained by the substrate quality for which the smoothing curve was applied. Data from each microbial community were separately used for the analysis to evaluate the difference among the 3 different microbial communities. We used the “mgcv” package for the additive model analysis [35]. The dependence of the individual lipids on the substrate quality was also tested using the additive model. To test the relationship between microbial community dissimilarity and substrate-quality dissimilarity, the Mantel test was performed using the “ecodist” package [36]. Substrate-quality dissimilarity is calculated as the difference in percentage of containing maple leaf between 2 substrates, whereas activity and community dissimilarity are calculated as the Euclidean distance between data matrices of decomposition activity (including soil respiration rate and 4 enzyme activities) and lipid profile, respectively. The decomposition activities were standardized before the dissimilarity analysis. The significance of Mantel coefficients, intercepts, and slopes were tested 999 times using bootstrap analysis. To explore the overall relationship between microbial decomposition activities, substrate quality, and microbial composition, we conducted redundancy analysis (RDA) with 5 soil decomposition activities as the explained variables and substrate quality and microbial lipid indicators as the explanatory variables. For RDA, lipid biomarkers that indicated the same microbial group were added in order to reduce the number of explanatory variables because, technically, the number of explanatory variables cannot exceed that of the sample size. The significance of the explanatory variables was tested using a permutation test (999 permutations). The RDA and permutation test were both conducted using the “vegan” package [37].

## Results

**Figure 1:**
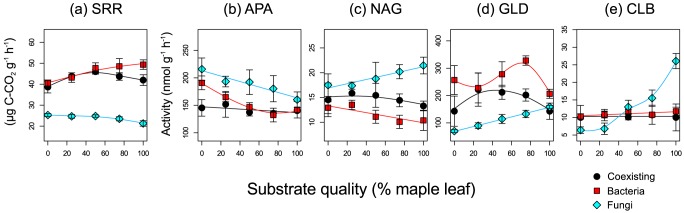
Effects of substrate quality on the soil respiration rate (SRR; a) and activities of acid phosphatase (APA; b), *N*-acetylglucosaminidase (NAG; c), β-d-glucosidase (GLD; d), and cellobiohydrolase (CLB; e). Each point indicates the mean value of 4 replicates in each treatment. The smoothing lines were drawn using the “mgcv” package of R [35]. Bars indicate 95% confidence interval. Black circles, red squares, and light-blue diamonds indicate the coexisting, bacterial, and fungal communities, respectively.

Soil respiration rates of the bacterial and fungal communities showed highly significant changes with variations in substrate quality (*P*<0.001; Table 2, Fig. 1a) but those of the coexisting community changed less significantly (*P*<0.01; Table 2, Fig. 1a). As the proportion of maple leaf increased, the soil respiration rates of the bacterial and fungal communities increased and decreased, respectively, but variations in the soil respiration rate of the coexisting community were relatively less explained by the variation in substrate quality (Table 2, Fig. 1a). Dependence of enzyme activity on the substrate quality in the coexisting community was weakest for acid phosphatase and *N*-acetylglucosaminidase and was similar to that in the bacterial community for β-d-glucosidase and cellobiohydrolase (Fig. 1, Table 2). In general, dependence of decomposition activities on the substrate quality in the coexisting community was weak compared with that in the bacterial or fungal community.

**Table 2:** The effects of substrate quality on microbial decomposition tested using separate additive models.

Soil microbial activity	Variation explained by substrate quality		
	Coexisting	Bacteria	Fungi
Soil respiration rate	65.0%**	67.4%***	73.7%***
Acid phosphatase	8.37%	79.4%***	50.8%***
*N*-Acetylglucosaminidase	15.9%	42.2%**	39.4%**
Glucosidase	**43.3%** *	**55.9%** *	90.6%***
Cellobiohydrolase	<0.001%	8.17%	94.6%***

* , **, and ***, indicate significance at *P*<0.05, 0.01, and 0.001, respectively. The statistical formula for this analysis was (decomposition activity)  = s (substrate quality) + residuals. The smoothing function was applied for the term enclosed by s() [35].

**Figure 2:**
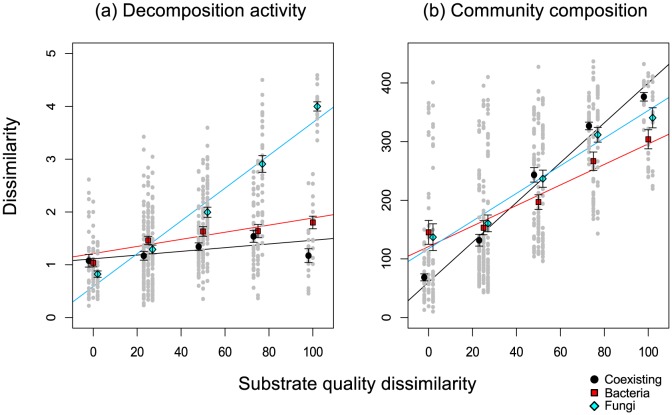
The relationships between substrate-quality dissimilarity and dissimilarity of decomposition activity (a) and microbial community composition (b). Substrate-quality dissimilarity is calculated as the difference between 2 substrates, whereas activity and community dissimilarity are calculated as the Euclidean distance between data matrices of activity and community composition, respectively. Black, red, and light-blue filled circles indicate the mean values of the coexisting, bacterial, and fungal communities, respectively. Grey circles indicate individual values. Black, red and light-blue solid lines indicate regression lines of the coexisting, bacterial, and fungal communities, respectively. Note that the points are slightly adjusted in the x-axis direction to distinguish the values of different microbial groups. Statistical results are shown in Table 3. Bars indicate standard errors of the mean.

To examine the stability of decomposition activity with variation in the substrate quality, we compared the dissimilarities in decomposition activity and those in substrate quality (Figs. 2a, Table 3). Dissimilarity indicates how the decomposition activities of the same community change against corresponding changes in substrate quality (see *Statistical analyses* in Materials and Methods). For the coexisting community, the overall decomposition activity was relatively stable (i.e., low dissimilarity), even when dissimilarity in the substrate quality increased. The dissimilarity in decomposition activity sharply increased with dissimilarity in substrate quality for the fungal community. For the bacterial community, the regression slope between activity dissimilarity and substrate-quality dissimilarity was not significantly different from that of the coexisting community, but the activity dissimilarity was, on average, higher than that of the coexisting community (Fig. 2a, Table 3).

**Table 3:** Results of regression analysis between dissimilarities in decomposition activity, lipid profile and substrate quality.

	Coexisting	Bacteria	Fungi
*Between decomposition activity and substrate quality* ^†^			
Mantel R	0.166	0.310	0.811
	(0.071–0.284)	(0.235–0.409)	(0.770–0.868)
Intercept	1.113	1.212	0.587
	(0.860–1.398)	(0.960–1.519)	(0.456–0.811)
Slope	0.0035	0.0067	0.0311
	(0.0016–0.0088)	(0.0039–0.0118)	(0.0270–0.0353)
*Between lipid profile and substrate quality* ^††^			
Mantel R	0.828	0.483	0.556
	(0.789–0.872)	(0.401–0.642)	(0.456–0.678)
Intercept	60.44	121.1	117.6
	(50.80–73.45)	(80.58–157.4)	(75.64–155.1)
Slope	3.395	1.750	2.355
	(3.074–3.746)	(1.663–2.988)	(1.690–3.639)

Values within parentheses indicate the 95% confidence intervals estimated by 999 bootstraps. The “ecodist” package of R was used to conduct these analyses. ^†^Corresponding to Fig. 2a. ^††^Corresponding to Fig. 2b.

To investigate the mechanism underlying the positive relationship between the coarse-scale taxonomic diversity (i.e., coexistence of fungi and bacteria) and stability of the decomposition activity observed in this experiment, the relationship between microbial lipid profile dissimilarity and substrate-quality dissimilarity was also analysed. We found that, in general, lipid profile dissimilarity increased with an increase in substrate-quality dissimilarity (Figs. 2b, Table 3). The regression slope was steepest for the coexisting community (Fig. 2b, Table 3). The slope coefficient for the fungal community was intermediate, although it was not significantly different from that for the coexisting community (Table 3). The slope coefficient of the bacterial community was significantly lower than that of the coexisting community (Table 3). Microbial biomass, not composition or physiology, can also be a controller of soil decomposition activities, but we did not find any difference in total lipid abundance (a proxy for microbial biomass) between the three microbial communities (Fig. S1).

Finally, the contributions of microbial composition (lipid profile) and substrate quality on microbial decomposition activity were simultaneously investigated using redundancy analysis (RDA) followed by a permutation test. For the coexisting community, gram-negative bacteria and protozoa were the microbial groups that significantly affected the decomposition activity, while the substrate quality did not affect the soil decomposition activity (Table 4). However, for the fungal and bacterial communities, substrate quality was the only significant factor that influenced the decomposition activity (Table 4).

**Table 4:** Effects of substrate quality and microbial groups on soil decomposition activities.

Explanatory variables	Proportion of explained variation		
	Coexisting	Bacteria	Fungi
Substrate quality	5.19%	35.5%***	65.5%***
General bacterial biomarker	3.80%	5.76%	0.937%
Gram-positive bacteria	8.97%	3.78%	0.939%
Gram-negative bacteria	16.9%**	2.37%	2.10%
Actinomycetes	1.75%	1.72%	3.80%
Arbuscular mycorrhizae	1.15%	2.12%	1.30%
Saprophytic fungi	5.20%	0.297%	4.39%
Protozoa	11.3%*	0.321%	1.63%
Residuals	45.7%	48.1%	19.4%

Each value indicates the proportion of explained variation by each factor, calculated using redundancy analysis (RDA) and a permutation test (999 permutations). All 5 decomposition activities were used as explained variables. Note that the sum of the biomarkers that indicate the same microbial group, and not individual biomarker lipids, was used here to reduce the number of explanatory variables in the RDA. Indicative biomarker lipids are specified in Materials and Methods. All variables were scaled before the RDA. *, **, and *** indicate significant effects at *P*<0.05, 0.01, and 0.001, respectively, by the permutation test.

## Discussion

In the microcosm experiment, we found that, in general, decomposition activities of the coexisting fungal-bacterial community were more stably maintained against variations in plant—substrate quality than those of either a fungal or bacterial community. The positive diversity–stability relationship found in this study is in accordance with what was found in the previous studies using aboveground systems [7], [8], [9] although cautions should be taken for interpretation of the results shown in this study because our manipulations of microbial community were still very rough. However, as there is currently no perfect method to control microbial community composition, we believe that our results provide an important first step for understanding the significance of microbial diversity in the stability of decomposition activity.

Previous studies also suggested that flexibility in community composition, a large variation in the abundance of each species in species-rich communities, can stabilize community-level function by compensating for environmental fluctuations [8], [12], [13], [14], which is thought to be the underlying mechanism of the positive correlations. To infer the underlying mechanism of the positive diversity–stability relationship found in this study, soil microbial lipids were analyzed in this study. Though there is a need for caution in interpretation of the lipid results [39], the lipid profile can be still used as a good proxy for microbial community composition and/or physiological state because a specific microbial group has a specific group of lipids in their cell membrane, and because sometimes lipids composition can be changed depending on microbial physiological state. According to the result of the dissimilarity analysis of the lipid profile, we also suggested that community composition or physiological state of the coexisting fungal-bacterial community can be more flexibly changed than that of either fungal or bacterial-dominated community. Therefore, similar to the previous studies [8], [12], [13], [14], flexible changes in the microbial community composition or physiological state could be the underlying mechanism of the observed diversity–stability relationship in the soil microbial community.

In addition to the dissimilarity analysis of the lipid profile, RDA result provided further supports for our suggestion that changes in community composition or physiological state could be the underlying mechanism of the observed diversity–stability relationship. The RDA result showed that, for the coexisting community, microbial groups significantly affected the decomposition activity, but only substrate quality was a significant factor that influenced the decomposition activity for the fungal and bacterial communities. One possible interpretation of the RDA result is that, for the coexisting community, substrate quality influenced the microbial composition, and in turn, the shift in the microbial composition buffered the control of substrate quality on the decomposition activity. However, in the case of the fungal or bacterial communities, compositional/physiological shift with changes in the substrate quality was less possible because either activities of bacteria or fungi were already inhibited by the selective biocides (i.e., the lack of bacterial or fungal function). Therefore, the effects of substrate quality cannot be buffered by compositional/physiological shifts, and only substrate quality was a significant factor that explained the decomposition activity. This buffering effect of the functionally/taxonomically diverse community has good accordance with the prediction from our recent theoretical model [4]. By comparing the coexisting fungal-bacterial community and communities dominated by either fungi or bacteria, our theoretical model demonstrated that the decomposition process in the coexisting community is less affected by the substrate quality (i.e., plant litter quality) than fungi- or bacteria-dominated community, and that this buffering effect is achieved by the flexible shift in microbial community composition [4].

In conclusion, our experiment demonstrated that the coexistence of fungi and bacteria stabilized soil decomposition activity, and a more flexible changes in composition and/or physiological state could explain the more stable decomposition activity in the coexisting community. Therefore, our study suggested that the previously proposed diversity–stability hypothesis [8], [12], [13], [14] could also be extended to the soil microbial community. We recognize that our experiment examined only a coarse-scale diversity–stability relationship, and that further methodological developments are obviously required for more comprehensive and detailed diversity–stability studies in the soil microbial community. However, our study still has an important implication that the functional diversity and community ecology of soil microbes should be incorporated into the context of a feedback loop between climate and ecosystem responses. Global climate change may alter the chemical quality of litter via a plant physiological response, or a shift in plant community composition, as well as subsequent decomposition processes and carbon cycling [40], [41], which in turn would induce further changes in climate. Taxonomic diversity of soil microbial communities, which could lead to the functional diversity of microbial communities, may attenuate the effects of changes in substrate quality on the decomposition process [42], and therefore, soil microbial community could work as a buffer in the climate–ecosystem feedback process.

## Supporting Information

Figure S1 **Fungi-to-bacteria ratio, Simpson's diversity index and the abundance of total lipids, bacterial lipids and a fungal lipid.**
(EPS)

Result S1 **Descriptions about fungi-to-bacteria ratio, Simpson's diversity index, lipid abundance in soil and references cited in the Supporting Information.**
(PDF)
