# Supplementary material for: A Coexisting Fungal-Bacterial Community Stabilizes Soil Decomposition Activity in a Microcosm Experiment
Source: PLoS One. 2013 Nov 18;8(11):e80320. doi: 10.1371/journal.pone.0080320 (PMC3832447; doi:10.1371/journal.pone.0080320)
Supplement: Result S1 — Descriptions about fungi-to-bacteria ratio, Simpson's diversity index, lipid abundance in soil and references cited in the Supporting Information. (PDF) [file pone.0080320.s002.pdf]

Supporting Results

Fungi-to-bacteria ratio, diversity index and lipid abundance in soil

To test the effect of selective biocides, fungi-to-bacteria ratio and Simpson's diversity index were calculated (Fig. S1a-b). Fungi-to-bacteria ratios in the fungal community (i.e., the bactericide treatment) were significantly higher than those in the coexisting community ($P <$ 0.05). This could indicate that the bactericide treatment inhibited the metabolic activity of bacteria, and that lipids of the killed bacteria were degraded immediately after the treatment. On the other hand, fungi-to-bacteria ratios in the bacterial community (i.e., the fungicide treatment) were not significantly lower than those in the coexisting community. One possible reason for the insignificant differences in the fungi-to-bacteria ratio between the bacterial and coexisting communities could be the fact that fungal cell debris contains relatively recalcitrant compounds such as chitin (Langley et al., 2003). Since decomposition activities of the two microbial communities were clearly different from each other (Fig. 1 and Table 2), the fungicide should have effects at least on the metabolic activities of fungal community in soil. Therefore, it is likely that a part of fungal debris (as well as the fungal lipid biomarker) remained in soil, and therefore, fungi-to-bacteria ratio as well as the fungal lipid abundance (Fig. S1e) did not change in the fungicide treatment. We also calculated Simpson's diversity index to test the effect of the biocides on lipid diversity extracted from the soil samples. Simpson's diversity index was calculated by using only the common lipid indicators that are listed in the main text. Since calculating diversity index for lipid data is not recommended as an index for microbial taxonomical diversity (Frostegård et al., 2011), we regarded Simpson's index simply as a parameter that represents the overall pattern of lipid profile. Simpson's index of the fungi-dominated community (i.e., the bactericide treatment) was significantly lower than those of the other two microbial communities (Fig. S1b). On the other hand, Simpson's index values of the coexisting and bacterial communities were not different each other. The insignificant difference in the diversity index between the coexisting and bacterial community could be again because of the presence of recalcitrant compounds in the fungal debris. Total lipid abundance, which is a rough proxy for microbial biomass in soil (Frostegård et al., 2011), was not different among the three microbial communities (Fig. S1c-e). This result indicated that microbial biomass was not a major factor driving the differences in decomposition activities among the three microbial communities.

References in Supporting Results

Frostegård, A., Tunlid, A., Bååth, E., 2011. Use and misuse of PLFA measurements in soils. Soil Biology and Biochemistry 43, 1621-1625.

Langley, J.A., Hungate, B.A., 2003. Mycorrhizal controls on belowground litter quality. Ecology 84, 2302-2312.
